# Mild encephalitis/encephalopathy with a reversible splenial lesion associated with systemic *Mycoplasma pneumoniae* infection in North America: a case report

**DOI:** 10.1186/s13256-022-03299-6

**Published:** 2022-02-20

**Authors:** Nafee T. Talukder, Ashlea Feezel, Jeremy E. Lankford

**Affiliations:** 1 Department of Neurology, Memorial Hermann Hospital, University of Texas Health Science Center at Houston, 6410 Fannin St, Ste 1014, Houston, TX 77030 USA; 2 Department of Pediatrics, Child Neurology, University of Texas Health Science Center at Houston, Houston, USA

**Keywords:** MERS, *Mycoplasma pneumoniae*, Pediatrics, Rare associations, Asia, North America

## Abstract

**Background:**

Mild encephalitis/encephalopathy with reversible splenial lesion is a clinical-radiological entity found to occur in the setting of an acute systemic inflammatory state with isolated lesions of the splenium of the corpus callosum and mild encephalopathy. Mild encephalitis/encephalopathy with reversible splenial lesion is commonly found to occur in children in the setting of viral infections. It has rarely been associated with *Mycoplasma pneumoniae* in the United States, unlike in Eastern and Southern Asia where this is much more prominent.

**Case presentations:**

A 5-year-old African-American boy with autism spectrum disorder presented to our emergency department with acute onset intractable vomiting, diarrhea, and abnormal tensing movements for 2 days, following a 6-day period of fatigue, fever, and spastic abdominal pain. Emergent work-up in our department ruled out acute gastrointestinal pathologies. Given the high fevers and encephalopathy, there was concern for meningitis or encephalitis. His cerebrospinal fluid profile was concerning for viral meningitis, however extensive infectious workup was negative. Magnetic resonance imaging of his brain demonstrated a T2 fluid-attenuated inversion recovery sequence hyperintensity in the splenium of the corpus callosum, read as postictal changes by radiology. Continuous video electroencephalography demonstrated mild diffuse encephalopathy without electrographic correlate of his tensing episodes. He was determined to have mild encephalitis/encephalopathy with a reversible splenial lesion in the setting of a postinfectious etiology. He was treated with a single pulse-dose of intravenous methylprednisolone, following which he gradually returned to his baseline the next day. Repeat magnetic resonance imaging and cerebrospinal fluid evaluation demonstrated resolution of previous findings. He was ultimately diagnosed with an acute *M. pneumoniae* infection, which was determined to be the etiology of his mild encephalitis/encephalopathy with a reversible splenial lesion.

**Conclusions:**

The presentation of mild encephalitis/encephalopathy with a reversible splenial lesion is often nonspecific, with behavioral symptoms ranging from irritability to disturbances in consciousness. Its prevalence is higher in the pediatric population, and is thought to be more of an infection-associated encephalopathy syndrome in this group. The infections are typically viral, more so than bacterial. *M. pneumoniae*, a small, atypical bacterium lacking a peptidoglycan cell wall, is a common respiratory tract pathogen found in children. Despite infection being so rampant in the pediatric community, very few cases of *M. pneumoniae*-associated mild encephalitis/encephalopathy with a reversible splenial lesion in the United States have been reported. In Eastern and Southern Asian countries, however, *M. pneumoniae*-associated mild encephalitis/encephalopathy with a reversible splenial lesion is much more commonly reported. This difference may potentially lie in the prevalence of macrolide-resistant *M. pneumoniae*, which is significantly higher in Asian countries given more liberal antibiotic use in *M. pneumoniae* infections. Infections with macrolide-resistant *M. pneumoniae* are reportedly greater in severity and duration. This amplified state may suggest a correlation between intensity of inflammatory response and the development of mild encephalitis/encephalopathy with a reversible splenial lesion. Given the rarity of *M. pneumoniae*-associated mild encephalitis/encephalopathy with a reversible splenial lesion in the United States, much remains unknown regarding predilection and optimum treatment strategy. As rates of macrolide-resistant *M. pneumoniae* begin to rise in the United States, maintaining a high level of suspicion remains key in better understanding this unique phenomenon.

## Background

Mild encephalitis/encephalopathy with a reversible splenial lesion (MERS) is a clinical–radiological entity found to occur in the setting of an acute systemic inflammatory state, with isolated lesions of the splenium of the corpus callosum (SCC) on brain magnetic resonance imaging (MRI) and in a mild encephalopathic state [1]. Most commonly seen in children, the presentation is generally nonspecific, and can range from irritability to alterations of consciousness [2]. The most common etiologies are thought to be related to seizures, metabolic changes, or infections. Most cases are associated with viral infections [3]. While cases linked to bacterial infections have been found in the literature, only a few cases of MERS associated with *Mycoplasma pneumoniae* (MP) have been reported. Of those found in the literature, most are reported in Eastern Asian countries [4], with only a handful reported in North America [1, 3]. MP is an atypical bacterium responsible for a significant proportion of respiratory tract infections in children [5], and is found to be responsible for up to 10% of pediatric encephalitis cases [6]. Though ubiquitous in the community, only a handful of MP-associated MERS cases have been reported in the United States. We present the case of a 5-year-old boy with autism spectrum disorder (ASD) in the United States presenting with intractable vomiting, diarrhea, and frequent whole-body tensing in the setting of MP-associated MERS.

## Case presentation

**Fig. 1:**
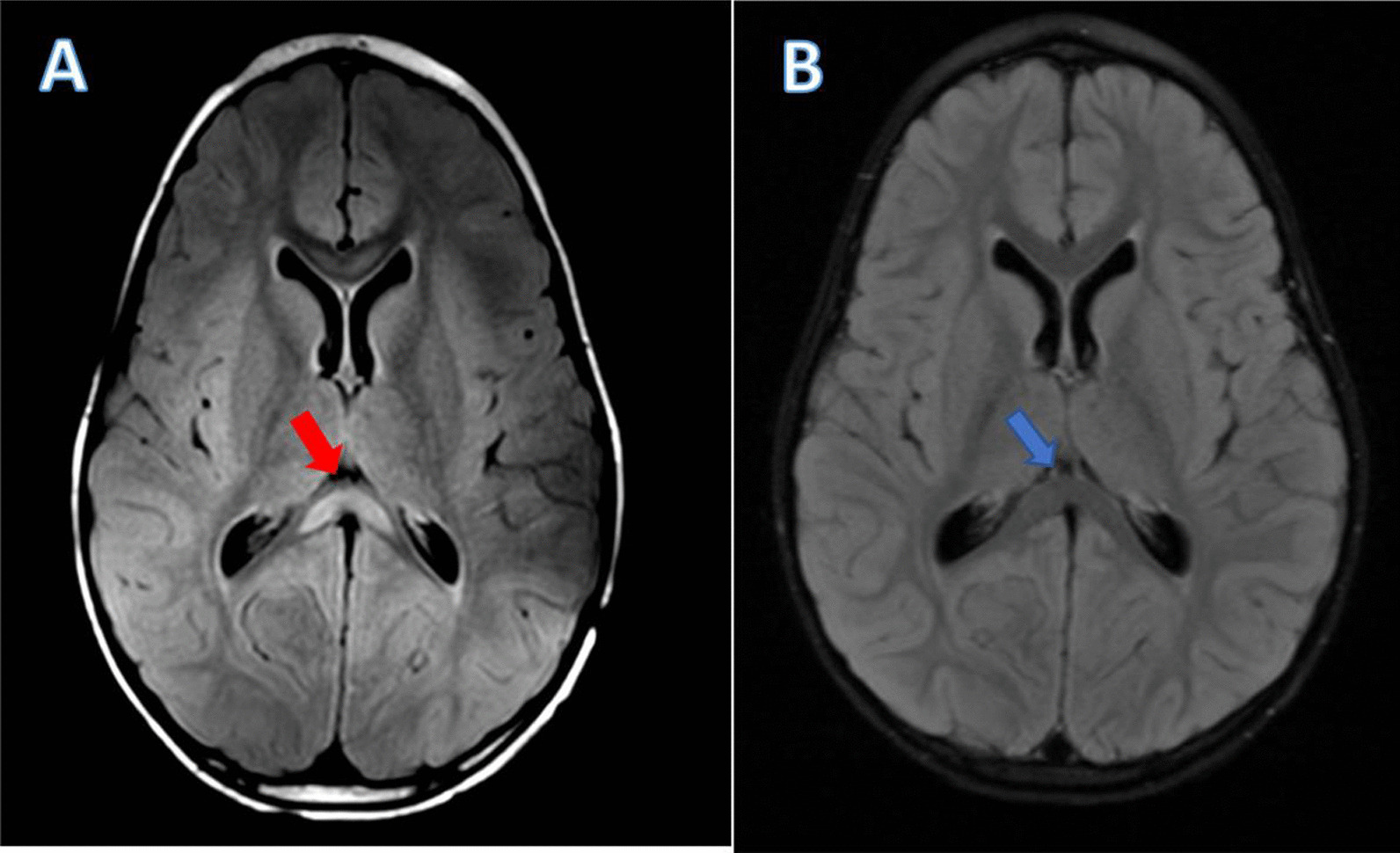
Neuroimaging demonstrating splenium of corpus callosum hyperintensity. Brain magnetic resonance imaging T2 fluid-attenuated inversion recovery sequence demonstrating **A** hyperintensity involving the splenium of the corpus callosum (red arrow), aka the “Boomerang Sign” [16], with **B** resolution of hyperintense signal on repeat imaging after 5 days (blue arrow) since initial imaging, and 2 days after pulse-dose intravenous steroids.

A 5-year-old African-American boy with autism spectrum disorder (ASD) and speech delay presented to our emergency department (ED) with decreased oral intake, in the setting of intractable vomiting and diarrhea along with abnormal movements for the past 2 days. As per his mother, he initially began to display symptoms of fatigue, fever, and spastic abdominal pain 6 days ago, shortly after these same symptoms occurred in his cousin and in his younger brother, who presented with a simple febrile seizure and was discharged home from the ED without further neurologic changes. They initially presented to an outside ED 6 days ago, where he was diagnosed with viral gastroenteritis and discharged home. However, over the past 2 days, the patient had become more somnolent with generalized weakness, and developed over 20 episodes of full-body tensing, with bilateral upper extremity flexion and lower extremities extension lasting 5–10 seconds per episode. His mother felt as if these were temporally correlated with his spastic abdominal pain, as he was screaming out crying during these episodes. She additionally noted mild improvement with over-the-counter antipyretics, however, he remained lethargic and irritable. Upon arrival at our ED, he was agitated, with generalized weakness, refusing to move, with a fever of 102 F. Emergent evaluation in pediatric surgery with abdominal X-ray, ultrasound, and MRI ruled out acute surgical abdomen, intussusception, or appendicitis. Infectious disease was initially consulted given concern for encephalitis or meningitis in the setting of fevers, intractable vomiting, and increased irritability and lethargy. He was started on empiric meningitic doses of vancomycin, ceftriaxone, and acyclovir. A brain MRI obtained by his primary team noted an isolated SCC hyperintensity on T2 fluid-attenuated inversion recovery (FLAIR) sequence (Fig. 1A), read as possible postictal changes by radiology. Cerebrospinal fluid (CSF) profile from lumbar puncture (LP) demonstrated a neutrophilic pleocytosis with elevated protein and normal glucose, concerning for possible viral meningitis.

Pediatric neurology was consulted regarding encephalopathy with reported abnormal repetitive movements, MRI changes, and CSF with inflammatory profile. Upon examination, he was lethargic, agitated, and refusing to change positions. Forty-eight hours of continuous video electroencephalogram (cvEEG) was completed, which was only notable for mild diffuse encephalopathy. Multiple brief tensing episodes were captured along with three brief desaturation episodes, all of which were without electrographic correlation, however, they did correlate with the patient screaming and clenching his stomach. Thus, the most likely etiology of his fits was determined to be pain-related, rather than seizures. Results from his infectious workup slowly began to return. CSF cultures, *Streptococcus pneumoniae* antigen, enterovirus, and herpes simplex virus (HSV) polymerase chain reaction (PCR) were negative; and his empiric acyclovir was discontinued after 2 days of treatment. In addition, viral respiratory PCR testing, COVID-19 nucleic acid amplification (NAA), blood cultures, and stool studies were all negative for the identification of the pathogen. Serological studies were sent soon after admission, with tests for Epstein–Barr virus, human immunodeficiency viruses (HIV), *Bartonella henselae*, and *Bartonella quintana* all negative prior to discharge.

Multidisciplinary discussions determined the patient likely had MERS in the setting of postviral, or postinfectious, systemic inflammatory changes. Based on literature review, the decision was made to administer a one-time pulse-dose of intravenous methylprednisolone at 20 mg/kg. The patient demonstrated gradual improvement in his mental status and movement following this corticosteroid dose; he began to eat more and, ultimately, returned back to his neurologic baseline over the next 24 hours. A repeat MRI brain and LP 5 days after his initial presentation and imaging demonstrated complete resolution of the SCC lesion (Fig. 1B) and inflammation in the CSF. His mother endorsed he was back to his neurologic baseline with only a mild decrease in oral intake due to his oral thrush. He was discharged home with home physical therapy (PT) and close outpatient follow-up. He completed 5 days of glycopeptide and beta-lactam antibiotics in-patient, and was discharged home without antibiotics as he had returned back to baseline and remained without fever, vomiting, or diarrhea. Following discharge, the patient’s remaining serological labs returned, which demonstrated an acute MP infection with elevated immunoglobulin (Ig)G (2.28 U/mL, normal < 0.9 U/mL) and IgM antibody (786 U/mL, normal < 770 U/mL).

## Discussion

Since its initial description in 2004 by Tada *et al*., based on a case series of 15 patients presenting with encephalitis/encephalopathy with isolated lesions in the SCC on brain MRI [1], multiple cases of MERS have been reported in the literature; yet much remains unknown. Prevalence is seemingly higher in the pediatric population, though multiple cases have been reported in adults as well. In the pediatric population, MERS is thought to be more of an infection-associated encephalopathy syndrome [7]. Associations with other systemic conditions such as metabolic derangements, such as hyponatremia and hypoglycemia, postictal changes, withdrawal of anti-seizure medications, high-altitude cerebral edema, and immune-mediated systemic inflammatory disorders such as systemic lupus erythematosus, have been reported more in adults [1, 2, 7, 8]. There are no known associations of direct central nervous system (CNS) infection or inflammation with the occurrence of MERS; rather, these changes are more likely to be caused by inflammatory changes in the setting of non-CNS infections [2]. Its presentation is often nonspecific, with the most common presenting neurological symptoms involving delirious behaviors, disturbances in consciousness, irritability, and even seizures [1, 3, 4].

The most common causative infectious organisms associated with MERS are viruses, such as influenza A and B, rotavirus, adenovirus, respiratory syncytial virus (RSV), and HSV, with one documented case associated with COVID-19 infection [2, 7, 9]. Bacterial etiologies are reported less commonly, although reports of MERS in association with *Escherichia coli* O-157, *Salmonella typhi*, *Streptococcus pneumoniae*, and Legionella have been reported [1, 3, 9, 10]. Our patient was ultimately diagnosed with MERS associated with a systemic MP infection. MP is an atypical bacterium with the inability to synthesize peptidoglycan cell walls; thus, confers natural resistance against beta-lactam and glycopeptide antibiotics, such as cephalosporins and vancomycin, respectively, as they specifically target cell wall biosynthesis [11–13]. This small, atypical bacterium is ubiquitous in the community and is a common respiratory tract pathogen affecting children. It is responsible for up to 40% of community-acquired pneumonia cases in children between the ages of 5–14 years [5], and up to 10% of pediatric encephalitis cases [6]. Additionally, symptoms can vary in children, with those older than 5 years of age more commonly presenting with fever, chills, sore throat, and progressive cough; while children 5 years old and younger can present with atypical symptoms, such as the vomiting and diarrhea seen in our patient [14]. Despite infection being so rampant in the pediatric community, very few cases of MP-associated MERS in the United States have been reported relative to Eastern and Southern Asian countries such as China, Japan, and India [3, 10]. This may, in part, be explained by the discrepancy in the prevalence of macrolide-resistant MP (MRMP) between these two regions, with a resistance rate of up to 80–90% in Eastern Asia compared with about 10% in the United States [4, 14]. Infections with MRMP have been reported to be greater in severity and longer in duration, and this amplified state may suggest a correlation between intensity of inflammatory response and MERS [4]. Given the rarity of MP-associated MERS reported in the United States, much remains unknown regarding its presentation, predilection, and treatment strategy.

Overall, MERS carries an extremely favorable prognosis, with complete resolution of symptoms between 3 and 19 days for type 1 MERS with lesions limited to the SCC, and up to 3–6 months in patients with type 2 MERS, which involves the entirety of the corpus callosum [5, 10]. The lack of controlled trials assessing effectiveness of therapeutic models and our limited understanding of the mechanisms underlying CNS involvement of MP-associated MERS has led to rather elusive treatment strategies. When associated with systemic infection, MERS is thought to result from an immune-mediated reaction of the body to the inciting pathogen [6].

This raises questions as to if solely treating the underlying pathogen is by itself sufficient, if immunomodulatory therapy is curative or beneficial, or if the syndrome itself self-resolves spontaneously. Our patient was treated with 5 days of beta-lactam and glycopeptide antibiotics without macrolide, fluoroquinolone, or tetracycline antibiotic treatment, which are considered first-line agents for MP [14]. Despite inadequate microbial coverage, our patient demonstrated complete resolution of his symptoms, imaging changes, and inflammation in the CSF within 5 days upon admission, about 11 days since initial symptom onset. His infection was untreated from a pharmacotherapeutic perspective; thus, this outcome questions the need for antibiotics in the management of MERS. The effect of antibiotics in MP infections overall remains a controversial topic based on prospective studies and retrospective analysis during epidemics of MP [4]. They are often self-limiting; however, they continue to be treated routinely with antibiotics. While antibiotics may have limited effects on MP infection as a whole, early corticosteroid use in cases of pneumonia due to MP has demonstrated a reduction in morbidity and mortality and prevention of severe disease progression [4]. The lack of controlled trials or systematic reviews has led to ambiguity in the approach to treating MP-associated encephalitis, and no standardized management strategy or guidance exists at this present time. Similar to MERS, this entity is thought to have an immunological origin. Several immunomodulatory therapeutic measures have been reported for treatment, including intravenous immunoglobulin, plasmapheresis, and corticosteroids [6]. All patients in these reported cases, regardless of treatment strategy, demonstrated a full recovery and reversal of MRI signal changes [4, 6, 15]. While prospective and retrospective analyses demonstrate overall benefit from corticosteroid use in pneumonia due to MP, use of systemic steroids have often been proposed for treating extrapulmonary manifestations, particularly conditions involving the central nervous system; however, data is limited on its potential benefits [15].

Our patient demonstrated complete resolution following inadequate antimicrobial coverage and a single dose of pulse-dose steroids, raising the question of whether our therapy was the driving force for his recovery. If so, would our findings suggest that sole corticosteroid therapy is sufficient to mitigate the inflammatory effects secondary to MP-associated MERS? Would our patient have recovered in due time without any intervention, and does corticosteroid therapy affect rate of recovery?

## Conclusions

The self-limited nature of MP infections and the lack of standardized controlled studies underlie our limitations in the approach of managing these conditions. In essence, much remains unknown regarding the management of this condition. Further studies in the United States remain a challenge given the limited number of cases reported for MP-associated MERS. As antibiotic use, or even overuse, continues, rates of macrolide resistance in MP will continue to rise in the United States; maintaining a high index of suspicion and a keen sense of awareness will remain key to unlocking further understanding of this unique phenomenon.

**Table of contents summary**

A 5-year-old boy with intractable vomiting and whole-body fits found to have mild encephalopathy with a reversible splenial lesion (MERS) due to systemic *Mycloplasma pneumoniae* infection; a rare etiology in North America, unlike in Eastern Asia.

## Data Availability

Not applicable to this manuscript type.

## Abbreviations

ASD: Autism spectrum disorder
CNS: Central nervous system
CSF: Cerebrospinal fluid
cvEEG: Continuous video electroencephalogram
ED: Emergency department
FLAIR: Fluid-attenuated inversion recovery
HSV: Herpes simplex virus
LP: Lumbar puncture
MERS: Mild encephalitis/encephalopathy with a reversible splenial lesion
MP: *Mycoplasma pneumoniae*
MRI: Magnetic resonance imaging
NAA: Nucleic acid amplification
PCR: Polymerase chain reaction
PT: Physical therapy
SCC: Splenium of the corpus callosum
